# Interaction of surfactants with barley leaf surfaces: time-dependent recovery of contact angles is due to foliar uptake of surfactants

**DOI:** 10.1007/s00425-021-03785-z

**Published:** 2021-11-26

**Authors:** Johanna Baales, Viktoria V. Zeisler-Diehl, Yaron Malkowsky, Lukas Schreiber

**Affiliations:** 1 Institute of Cellular and Molecular Botany, Department of Ecophysiology, University of Bonn, Kirschallee 1, 53115 Bonn, Germany; 2 Nees Institute for Biodiversity of Plants, University of Bonn, Meckenheimer Allee 170, 53115 Bonn, Germany

**Keywords:** Cuticle, Diffusion, Epicuticular wax crystallites, Hydrophobicity, Transport, Wetting

## Abstract

**Main conclusion:**

Time-dependent contact angle measurements of pure water on barley leaf surfaces allow quantifying the kinetics of surfactant diffusion into the leaf.

**Abstract:**

Barley leaf surfaces were sprayed with three different aqueous concentrations (0.1, 1.0 and 10%) of a monodisperse (tetraethylene glycol monododecyl ether) and a polydisperse alcohol ethoxylate (BrijL4). After 10 min, the surfactant solutions on the leaf surfaces were dry leading to surfactant coverages of 1, 10 and 63 µg cm^−2^, respectively. The highest surfactant coverage (63 µg cm^−2^) affected leaf physiology (photosynthesis and water loss) rapidly and irreversibly and leaves were dying within 2–6 h. These effects on leaf physiology did not occur with the lower surfactant coverages (1 and 10 µg cm^−2^). Directly after spraying of 0.1 and 1.0% surfactant solution and complete drying (10 min), leaf surfaces were fully wettable for pure water and contact angles were 0°. Within 60 min (0.1% surfactant) and 6 h (1.0% surfactant), leaf surfaces were non-wettable again and contact angles of pure water were identical to control leaves. Scanning electron microscopy investigations directly performed after surfactant spraying and drying indicated that leaf surface wax crystallites were partially or fully covered by surfactants. Wax platelets with unaltered microstructure were fully visible again within 2 to 6 h after treatment with 0.1% surfactant solutions. Gas chromatographic analysis showed that surfactant amounts on leaf surfaces continuously disappeared over time. Our results indicate that surfactants, applied at realistic coverages between 1 and 10 µg cm^−2^ to barley leaf surfaces, leading to total wetting (contact angles of 0°) of leaf surfaces, are rapidly taken up by the leaves. As a consequence, leaf surface non-wettability is fully reappearing. An irreversible damage of the leaf surface fine structure leading to enhanced wetting and increased foliar transpiration seems highly unlikely at low surfactant coverages of 1 µg cm^−2^.

## Introduction

Plant protecting agents (AIs) are usually sprayed to leaf surfaces (Arand et al. 2018) which are covered by the wax-impregnated plant cuticle (Dominguez et al. 2017). Due to their hydrophobic nature, plant cuticles are non-wettable leading to contact angles of water of about 90° or higher (Taylor 2011). Leaf surfaces of many species, e.g. Poaceae, are classified as super-hydrophobic (Barthlott et al. 2016), since they are characterized by three-dimensional epicuticular wax crystallites leading to pronounced leaf surface roughness rendering leaf surfaces essentially non-wettable (Barthlott and Neinhuis 1997). Therefore, surfactants have to be added to spray solutions containing AIs, to enhance leaf surface wetting and adhesion of spray droplets (De Ruiter et al. 1990). Besides improved leaf surface wetting, surfactants have also been shown to act as plasticizers on the transport limiting barrier of the cuticle; thus, enhancing the diffusion of AIs into the leaf interior (Burghardt et al. 1998; Buchholz and Schönherr 2000; Hess and Foy 2000).

As amphiphilic compounds surfactants reduce the surface tension of water, thus, they allow wetting of hydrophobic surfaces (Adamson and Gast 1976) by aqueous solutions, and they form micelles in water (Schick 1987). This could negatively affect the structure and function of leaf surfaces. Micelles can solubilise hydrophobic lipids in water and it can be speculated that they might also be able to solubilise cuticular waxes (Tamura et al. 2001). Since waxes form the transport limiting barrier of the plant cuticle (Schreiber 2010), this could irreversibly damage and reduce barrier properties of leaf surfaces, which would be an unwanted side effect of surfactants application. As a consequence, transpiration might be enhanced (Räsch et al. 2018) and crops might be more drought sensitive. An improved wetting of leaf surfaces might lead to enhanced leaching of ions and solutes (Tukey 1970), since it will facilitate the formation of water films on leaf surfaces. This could lead to nutrient imbalances and it might also promote leaf surface colonization by microorganisms including potential plant pathogens (Marcell and Beattie 2002).

Two non-ionic surfactants, the polydisperse BrijL4 and the monodisperse tetraethylene glycol monododecyl ether (C_12_E_4_), were selected to study their interaction with barley leaf surfaces. Barley was chosen as model for grass species in general (crops as well as weeds) since its leaf surface is highly water repellent and characterized by a pronounced roughness due to epicuticular wax platelets (Jorgensen et al. 1995). BrijL4 was chosen as a typical example for technical polydisperse surfactants, which are frequently used in agrochemistry. Since its mean calculated chemical composition is given as C_12_E_4_, we intended to compare it with the monodisperse C_12_E_4_, which is a chemically pure (p.a.) substance.

It was our intention to investigate whether surfactants, applied to barley leaf surfaces and allowed to dry off, thus, resulting in different surface coverages, lead to structural and functional changes of the leaf surface and potentially result in irreversibly enhanced wetting of pure water, increased foliar transpiration or qualitative and/or quantitative changes in the wax layer. Therefore, time-dependent changes of contact angles of pure water after surfactant application were recorded, wax composition and amounts were measured by gas chromatography and mass spectrometry, epicuticular wax crystallites were visualized using scanning electron microscopy and rates of water loss were determined gravimetrically. A realistic surfactant concentration of 0.1%, normally used in agrochemical applications (Foster et al. 2004), was compared to 1% and 10% surfactant concentrations to identify a potential concentration dependence of the surfactant application. Our results presented in the following allow to conclude that microscopic structure and transport function of cuticles covering barley leaf surfaces are not persistently altered or damaged when they interact with alcohol ethoxylates applied at leaf surface concentrations at a realistic surfactant coverage of 1 µg cm^−2^ (0.1%), which is normally used in agrochemical applications.

## Materials and methods

### Chemicals

All chemicals used were of high analytical purity (p.a.). As model surfactants, non-ionic alcohol ethoxylates were used in the experiments. The monodisperse tetraethylene glycol monododecyl ether (C_12_E_4_; Sigma-Aldrich), composed of n-dodecanol (C_12_) and 4 ethylene oxide units (E_4_), was compared with the polydisperse BrijL4 (Sigma-Aldrich), since the calculated mean molecular composition of the technical surfactant BrijL4 is given as C_12_E_4_ as well. The cuticle water partition coefficient of the monodisperse C_12_E_4_, describing the lipophility of a molecule, is 6000 (Burghardt et al. 1998). There is no value available for the polydisperse BrijL4. However, since its mean chemical structure is given as C_12_E_4_ it can be assumed to have a similar lipophility as the monodisperse surfactant.

### Plant material, growth conditions and surfactant treatments of leaves

Barley plants were chosen for the experiments since their leaf surfaces are super-hydrophobic and thus nearly non-wettable (Barthlott et al. 2016). Barley seeds (*Hordeum vulgare* cv. Scarlett) were provided by the Institute of Plant Breeding (Bonn University). They were stratified at 4 °C for 1 week, and germinated in the dark at 25 °C on wet filter paper for 2 days. Subsequently, plants were cultivated in a growth chamber (16 h light period, 150 μmol m^−2^ s^−1^, day/night temperatures 23 °C/20 °C, relative humidity 50 to 65%) for another 12 days on soil (Einheitserde Typ 1.5, Nitsch, Kreuztal, Germany). Plants were watered twice a week with tap water and used for the experiments at the age of 14 days (2 days germination + 12 days growth). At this stage, 2 leaves had developed and the 2nd leaf was used for the experiments.

Surfactants were sprayed on the adaxial leaf surfaces at aqueous concentrations of 0.1, 1 and 10% (v/v) using an airbrush system (Start Single Action Airbrush-Pistol, Conrad, Bonn, Germany). Spraying was standardized (3 × 1 s, distance to the leaf surface 10 cm) in preliminary experiments, which ensured reproducible surfactant coverages after drying of the spray solutions. Visually the leaf surfaces covered with different surfactant amounts looked dry in equilibrium with the ambient air humidity of 52.3 ± 3.4% (own determination). Surfactant coverages on the leaf surfaces were 1, 10 and 63 µg cm^−2^, when using the corresponding surfactant concentrations of 0.1, 1 and 10% for spraying. Leaves were scanned for determination of the surface areas. Since all three surfactant solutions used were significantly above the critical micelle concentrations, they all had similar surface tensions of 30 ± 3.5 mN m^−1^ (average value for both surfactants and all concentrations calculated from own determinations). Visible stomatal infiltration of the leaves did not occur when sprayed with 0.1 and 1% surfactant solutions since this directly would lead to dark spots in the leaf due to changes of the light diffraction. With the 10% surfactant solution, this was partially visible.

### Quantification of surfactant coverages and wax amounts on leaves after spraying

To verify that homogeneous and reproducible surfactant coverages can be obtained after standardization of spraying with the air brush system, surfactant amounts sprayed on microscopic cover slips of glass and on barley leaf surfaces were quantified by gas chromatography. After drying off cover slides were extracted with chloroform (1 ml) spiked with tetracosane (10 µg per sample), serving as internal standard for quantification. Sprayed barley leaves were extracted in 15 ml chloroform directly after drying of the spray solution and subsequently after 1, 2, 3, 4 and 6 h. In addition, barley leaves which had been sprayed were washed with water three times after drying of the spray solution and extracted with 15 ml chloroform. Extracts (spiked with 10 µg tetracosanoic internal standard) were reduced to a final volume of 200 µl under a gentle stream of nitrogen at 60 °C.

Extracts were analysed using gas chromatography and mass spectrometry as described recently in detail (Baales et al. 2021). Prior to gas chromatography, samples were derivatized using BSTFA (*N*, Obis-(trimethylsilyl)trifluoroacetamide, Merck) at 70 °C for 45 min. Quantification was performed by on-column injection analysing 1 µl sample in a gas chromatograph connected to a flame ionization detector (GC-FID: Agilent 5980; column: 30 m DB-1 with an inner diameter of 0.32 mm and film 0.1 µm, Agilent). Amounts of detected surfactant and wax molecules were related to the sprayed and extracted areas (cover slides or barley leaves). Leaves were scanned for determination of the surface areas. Identification of wax and surfactant molecules was achieved by mass spectrometry (GC: Agilent 6890 N; MS: Agilent 5973 N mass selective detector; column: 30 m DB-1MS with an inner diameter of 0.32 mm and film 0.1 µm). Identification of the individual peaks was based on fragmentation patterns of the peaks and by comparing obtained mass spectra with stored mass spectra in a homemade library.

### Measurement of photosynthesis

A chlorophyll fluorometer (Junior-PAM, Pulse Amplitude Modulation; Walz, Effeltrich, Germany) was used for photosynthesis measurements. This non-invasive technique allows the non-destructive measurement of the electron transport rate in photosynthesis over time. Barley leaves, sprayed with different surfactant concentrations, leading to coverages of 1, 10 and 63 µg cm^−2^, were investigated. Leaves sprayed with pure water served as control. Leaf photosynthesis was monitored for 10 h and the photosynthetic yield was measured every 15 min. The intensity of the actinic light was 190 μmol m^−2^ s^−1^.

### Measurement of residual foliar transpiration

Residual transpiration of surfactant-treated barley leaves was determined by gravimetry (Sartorius CPA225D, Sartorius). Barley leaves were sprayed with different surfactant concentrations leading to a final coverage of 1, 10 and 63 µg cm^−2^. Untreated leaves served as a control. Fresh weights of 14-day-old detached leaves were initially measured, before time-dependent weight losses of leaves were measured. At the beginning (first hour) weight loss was recorded every 5 min and for the next 120 min it was recorded every 30 min. During transpiration measurements leaves were kept at 2% humidity over activated silica gel and at 25 °C to keep the driving force for transpiration constant and at maximum. Finally, dry weights of the leaves were obtained after drying them overnight at 60 °C. Permeances *P* (m s^−1^) were calculated using the following equation: *P* = *F*/(Δ*C*). *F* is the flow of water (g m^−2^ s^−1^) and ∆*C* (g m^−3^) represents the driving force (Niederl et al. 1998).

### Contact angle measurements

For contact angle measurements of pure water on freshly cut pieces of surfactant-treated barley leaves, samples were carefully placed on clean microscopic slides using a double-side adhesive tape. Droplets of pure water (10 µl) were carefully placed on the surfactant-treated leaf surfaces or on untreated leaves serving as control and detached from the needle. Each droplet equilibrated 10 s on the surface before measurement. Each measurement was done with a fresh water droplet. Contact angles were measured using a drop shape analyser, equipped with a video camera and connected to a computer (DSA 25E; Krüss, Hamburg, Germany). Contact angles were measured using the sessile drop method. This image analysis method for determining the contact angle from the shadow image of a sessile drop is based on an ellipse algorithm (tangent-1). Time-dependent changes of contact angles were measured on treated barley leaves at time intervals varying between 10 and 60 min up to 6 h. Measurements of contact angles were taken with unrelated leaf samples to ensure that always fresh and not dehydrated leaf material was used for contact angle measurements.

### Scanning electron microscopy (SEM)

Treated and untreated barley leaves were fixed to aluminium sample holders (diameter 2.5 cm, Plano Marburg, Germany) with double-side adhesive tape and dried over silica at 21 °C for at least 2 days. All samples were sputtered with gold (SCD 040, Balzers Union, Wiesbaden, Germany) at 25 mA for 30 s leading to a final coating thickness of about 25 nm. Scanning electron microscopy (SEM, Cambridge S200 Stereoscan, Cambridge Instruments, Cambridge UK, equipped with DISS5 image acquisition system, point Electronic, Halle, Germany) was performed at an accelerating voltage of 15 keV in high vacuum.

### Statistical analysis

Data analysis and statistical test were carried out with Origin Pro 9 (Origin Lab). Normal distribution of the data was tested with the Shapiro–Wilk test. Significant differences between means were tested with a one-way ANOVA (Fisher’s LSD) at a significance level of 0.05.

## Results

### Coverage of surfaces with surfactants

**Fig. 1:**
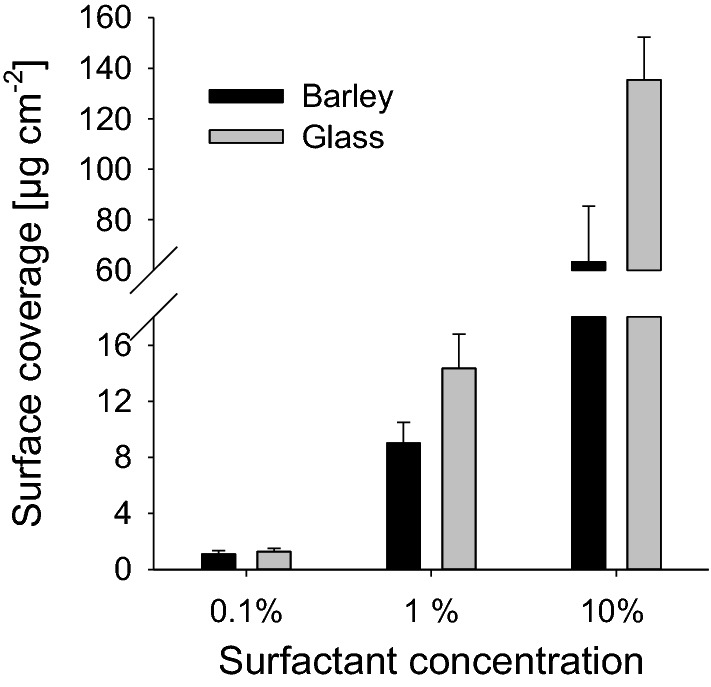
Amount of C_12_E_4_ (µg cm^−2^) deposited on barley leaf (black bars) and glass surfaces (grey bars) after spraying with 0.1, 1 and 10% aqueous solutions. Coverages with C_12_E_4_ were 1.2 ± 0.2; 9.0 ± 1.5 and 63.4 ± 22.0 µg cm^−2^ on barley leaf surfaces with increasing concentrations. Surfactant coverages were 0.8 ± 0.2; 14.4 ± 2.5 and 135.4 ± 16.9 µg cm^−2^ on glass surfaces with increasing concentrations. Data points represent means with standard deviations (*n* = 3)

On both surfaces a mean surfactant coverage close to 1 µg cm^−2^ (glass: 0.8 ± 0.2 µg cm^−2^; leaf: 1.2 ± 0.2 µg cm^−2^) was obtained with 0.1% surfactant concentration (Fig. 1). Concentrations of 1% resulted in a surfactant coverage of about 10 µg cm^−2^ on barley (9.02 ± 1.48 µg cm^−2^), whereas on glass it was 14.35 ± 2.45 µg cm^−2^ (Fig. 1). 10% solutions lead to a surface coverage of 63.42 ± 22.01 µg cm^−2^ on barley and 135.38 ± 16.93 µg cm^−2^ on glass. Comparable results were obtained for BrijL4. To keep it simple, in the following we will always refer to leaf surface coverages of 1and 10 µg cm^−2^ for the 0.1% and 1.0% surfactant solutions, respectively.

### Effects of surfactants on photosynthesis and residual transpiration

**Fig. 2:**
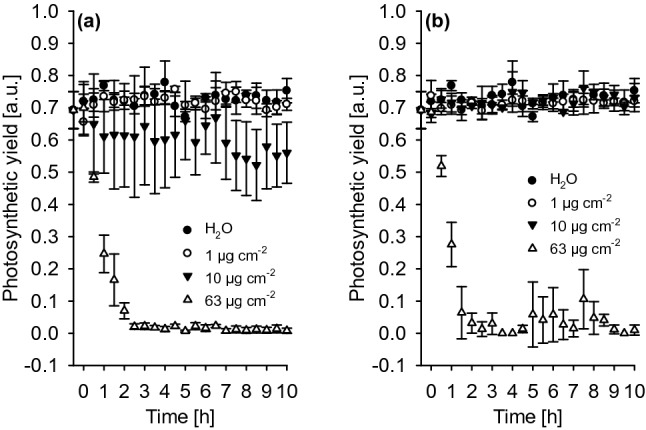
Photosynthetic yield of barley leaves measured over 10 h after surfactant treatment with C_12_E_4_ (**a**) and BrijL4 (**b**) leading to final surfactant coverages of 1 (white circles), 10 (black triangles) and 63 µg cm^−2^ (white triangles). Controls (black circles) were measured after spraying pure water. Data points represent means with standard deviations (*n* ≥ 3)

Photosynthetic yield was not affected within 10 h at surfactant coverages of 1 µg cm^−2^ (Fig. 2a, b). At 10 µg cm^−2^ C_12_E_4_ leads to a slightly lower yield of 0.6 compared to 0.7 of the control (Fig. 2a), whereas BrijL4 did not affect the photosynthetic yield at this surfactant coverage (Fig. 2b). The next day (about 24 h after surfactant application), few small spots of tissue necrosis detectable were observed on the leaves treated with 10 µg cm^−2^ surfactant, this was never observed with surfactant coverages of 1 µg cm^−2^. A complete inhibition of photosynthesis could be observed with both surfactants at the highest coverages (ca. 63 µg cm^−2^) already 2 h after treatment. There was no recovery within 10 h, since leaves were dead (Fig. 2a, b).

**Fig. 3:**
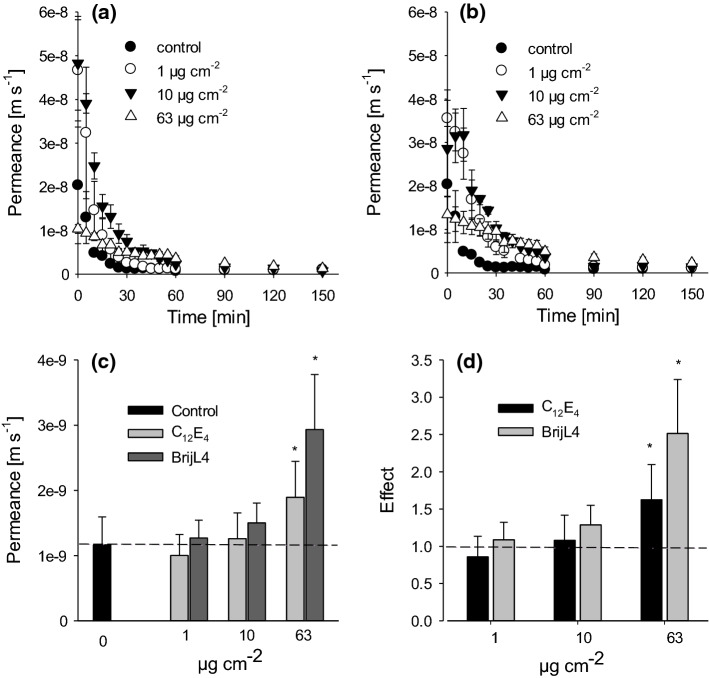
Residual (cuticular) transpiration of detached barley leaves after surfactant treatment leading to final surfactant coverages of 1 (white circles), 10 (black triangles) and 63 µg cm^−2^ (white triangles). Controls (black circles) were measured after spraying pure water. Time-dependent permeances after C_12_E_4_ (**a**) and BrijL4 (**b**) treatments indicate a constant residual transpiration after 60 to 90 min with 1 and 10 µg cm^−2^ surfactant coverage. Mean constant residual permeances (**c**) and effects of surfactants (**d**) on permeances with dotted lines indicating the control values for the permeance (**c**) and the effect (**d**). Data points (**a**, **b**) represent means with standard deviation (*n* ≥ 3). Bars (**c**, **d**) represent means with standard deviation (*n* ≥ 9). Asterisks indicate differences between treatments and control at significance levels of 0.05(*) applying a one-way ANOVA

Residual transpiration measurements with detached leaves after spraying with 0.1% and 1% surfactant solutions showed a constant decrease of permeances within the first 30 to about 90 min, which describes the continuous closure of stomata (Fig. 3a, b). Residual permeances were constant between 90 and 150 min with the 0.1% and 1% surfactant solutions and this describes the residual cuticular transpiration with closed stomata. With the 10% surfactant solution residual permeances continued to decrease for 150 min (Fig. 3a, b). Permeances of the control leaves were not significantly different from leaves covered with 1 and 10 µg cm^−2^of C_12_E_4_ or BrijL4, whereas at coverages of 63 µg cm^−2^, permeances were significantly higher compared to control leaves (Fig. 3c). At surfactant coverages of 63 µg cm^−2^, the effects of the surfactants on transpiration were 1.5-fold for C_12_E_4_ and 2.4-fold for BrijL4 (Fig. 3d).

### Chemical changes of epicuticular waxes

**Fig. 4:**
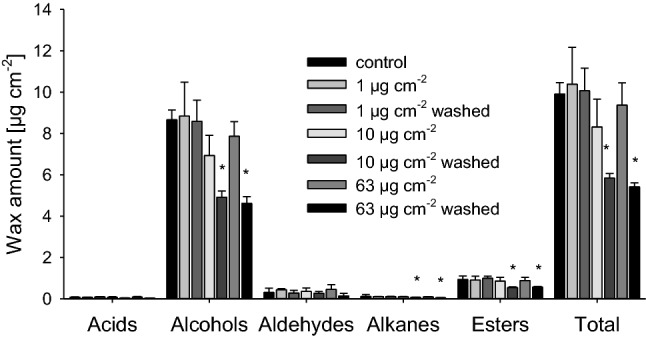
Substance classes and total wax amounts (µg cm^−2^) of barley leaf surfaces after C_12_E_4_ treatment leading to final surfactant coverages of 1, 10 and 63 µg cm^−2^. Five major substance classes (acids, alcohols, aldehydes, alkanes and esters) were identified. Total wax amount of control leaves was 9.9 ± 0.6 µg cm^−2^. Wax amounts determined after surfactant treatment without subsequent washing with pure water did not change. Wax amounts were lower with 10 and 63 µg cm^−2^, when surfactants were washed off again after spraying and drying with pure water. Bars represent means with standard deviation (*n* = 3). Asterisks indicate differences between treatments and control at significance levels of 0.05(*) applying a one-way ANOVA

Cuticular wax of barely is composed of five main substance classes: acids, alcohols, alkanes, aldehydes and esters (Fig. 4). The most abundant component is hexacosanol (C_26_ alcohol), which contributes to about 85% of total cuticular wax. Total wax amounts of barley leaves were about 9.9 ± 0.6 µg cm^−2^. There were no quantitative and qualitative changes in substance classes or individual wax components after treating leaf surfaces with both surfactants and all three surfactant loads (Fig. 4). Rinsing the surfaces with water after surfactant treatment led to a significant loss of total wax amount per unit leaf surface area with surfactant loads of 10 and 63 µg cm^−2^, respectively. The amounts of alcohols, alkanes and esters decreased significantly.

### Structural changes of epicuticular waxes

**Fig. 5:**
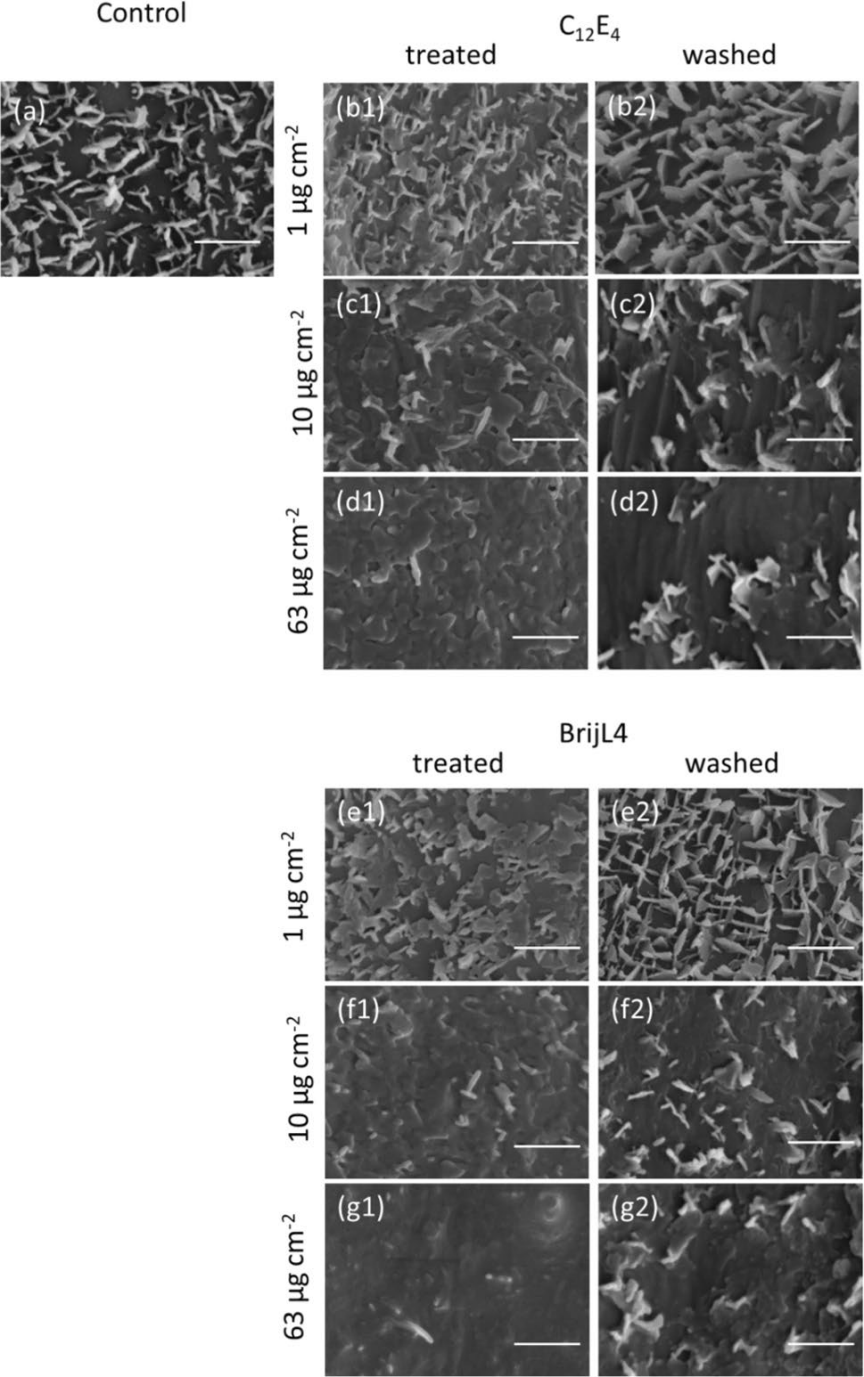
Scanning electron microscopic images of barley leaf surfaces at different surfactant coverages (1, 10 and 63 µg cm^−2^) with C_12_E_4_ (**b1**, **c1**, **d1**) and BrijL4 (**e1**, **f1**, **g1**) after surfactant drying and after subsequent washing off dried C_12_E_4_ (**b2**, **c2**, **d2**) and dried BrijL4 (**e2**, **f2**, **g2**). Leaf surfaces treated with pure water (**a**) and again washed with pure water after drying served as control. Bars = 2 µm

Scanning electron microscopic (SEM) investigations showed that barley surface waxes are arranged as platelets with an average height of 1 µm (Fig. 5a). After spraying with increasing surfactant concentrations wax platelets became more and more invisible (Fig. 5b1, c1, d1, e1, f1, g1). After washing leaf surfaces with water, three-dimensional wax crystallites completely reappeared at surfactant coverages of 1 µg cm^−2^ (Fig. 5b2, e2). With leaves treated with 10 and 63 µg cm^−2^ surfactant concentration three-dimensional wax crystallites only partially recovered after washing (Fig. 5c2, d2, f2, g2).

### Contact angle measurements

**Fig. 6:**
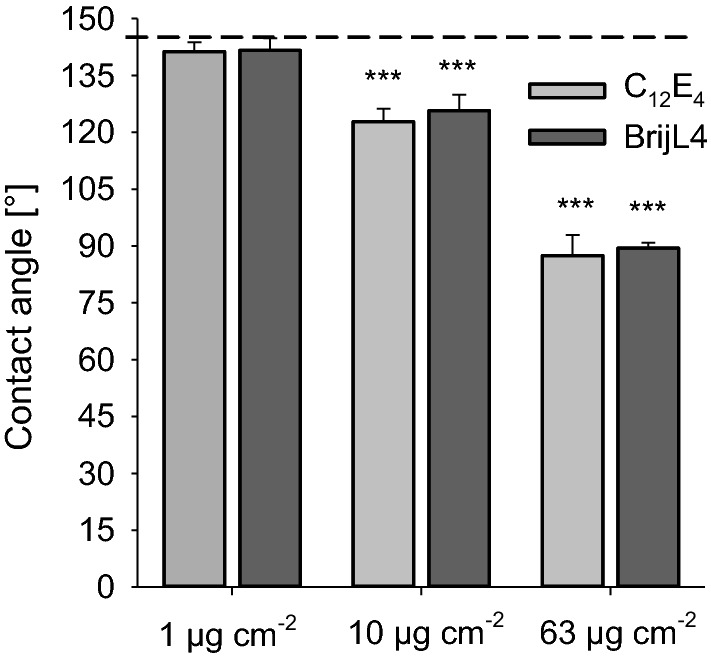
Contact angles of pure water droplets on barley leaf surfaces with three different surfactant coverages (1, 10, 63 µg cm^−2^) after treatment with C_12_E_4_ (grey bars) and BrijL4 (black bars). After drying, surfactants were washed off again before contact angles were measured. The dashed line gives the contact angle (144° ± 3°) of barley leaves which were treated and washed with pure water. Contact angles of barley leaves treated with 1 µg cm^−2^ surfactant did not change, whereas they decreased after treatments with 10 and 63 µg cm^−2^. Bars represent means standard deviation (*n* ≥ 3). Asterisks indicate differences between treatments and control at significance levels 0.001 (***) applying a one-way ANOVA

The contact angle of a 10 µl water droplet on an untreated barley leaf was 144 ± 3° (dashed line in Fig. 6). After drying of leaf surfaces, which were sprayed with the two surfactants and the three different concentrations (0.1, 1 and 10%), contact angles of pure water were 0° since droplets spread fully. When rinsing the leaf surfaces with pure water, after they had been sprayed with the different surfactant solutions and fully dried off in 10 min, contact angles higher than 0° could be measured and leaves were again non-wettable (Fig. 6). At a surfactant coverage of 1 µg cm^−2^ (both surfactants) contact angles fully recovered to 144°. Surfactant loads of 10 µg cm^−2^ surfactant led to a reduced contact angle of ca. 125°, surfactant loads of 63 µg cm^−2^ led to contact angles of only 90° (Fig. 6).

### Time-dependent recovery of contact angles after surfactant treatments

**Fig. 7:**
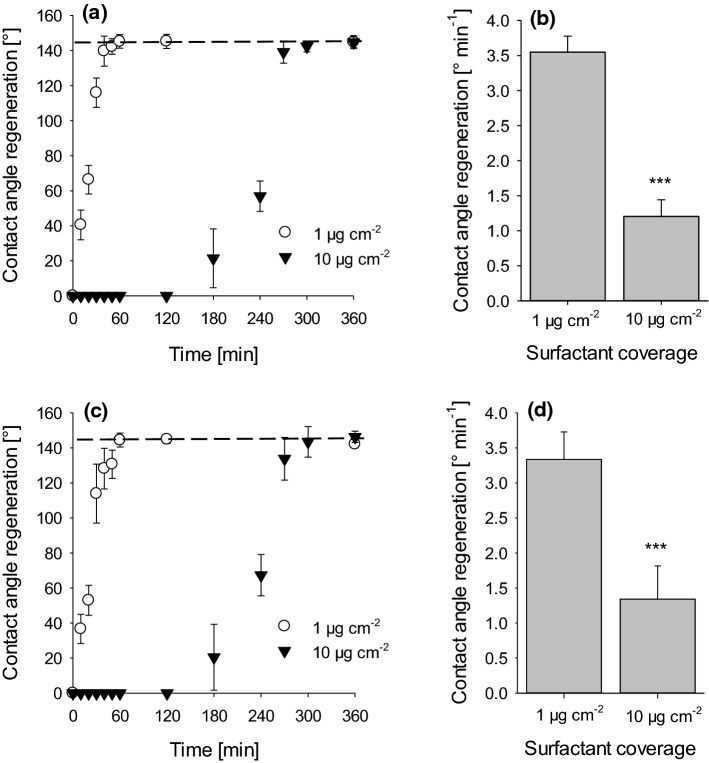
Time-dependent contact angle measurements of water on barley leaf surfaces after treatment with C_12_E_4_ (**a**) and BrijL4 (**c**) leading to two different surfactant coverages of one (white circle) and 10 µg cm^−2^ (black triangle). Contact angles were measured after drying of sprayed surfactant solution. Contact angles (144° ± 3°) of controls (dashed lines) represent barley leaves treated with pure water. Rate constants of contact angle recovery (° min^−1^) after treatment with C_12_E_4_ (**b**) and BrijL4 (**d**) and 2 different surfactant coverages of 1 and 10 µg cm^−2^. Data points and bars represent means with standard deviation (*n* ≥ 3). Asterisks indicate differences between treatments and control at significance levels 0.001 (***) applying a one-way ANOVA

Time-dependent recovery of contact angles of water droplets was measured after drying of leaves sprayed with 0.1 and 1% surfactant solutions. At surfactant coverages of 1 µm cm^−2^ (0.1% surfactant solution), contact angles fully recovered with both surfactants after 30 to 60 min (Fig. 7a, b). With the higher surfactant loads of 10 µg cm^−2^ (1% surfactant solution) leaves stayed completely wettable for nearly 2 h before contact angles fully recovered within the next 2–4 h (Fig. 7a, b).

The speed of contact angle recovery was calculated from regression lines fitted to those parts of Fig. 7a, c where contact angles were continuously increasing again (between 0 and 40 min for a coverage of 1 µg cm^−2^ and between 3 and 5 h for a coverage of 10 µg cm^−2^). Slopes of the regression lines were about three-times steeper (3 to 3.5° min^−1^; Fig. 7b) at 1 µg cm^−2^ surfactant coverage compared to 10 µg cm^−2^ surfactant coverage (1.2 to 1.3° min^−1^; Fig. 7d).

### Time-dependent reappearance of epicuticular wax crystallites after surfactant treatments

**Fig. 8:**
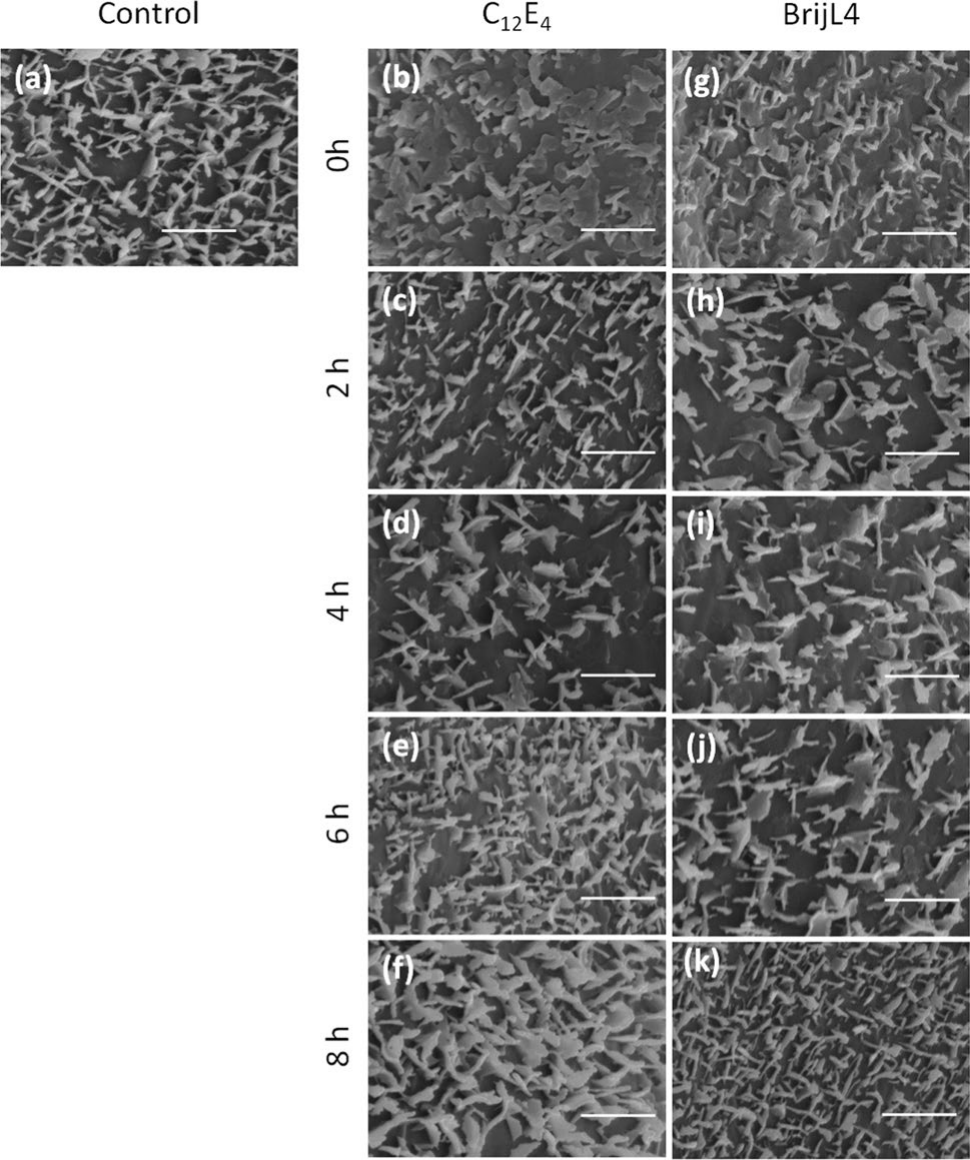
Scanning electron microscopic images of barley leaf surfaces at increasing times (0, 2, 4, 6 and 8 h) after treatment with C_12_E_4_ (**b**, **c**, **d**, **e**, **f**) and BrijL4 (**g**, **h**, **i**, **j**, **k**) leading to a surfactant coverage of 1 µg cm^−2^ in comparison to control leaves treated with pure water (**a**). Bars = 2 µm

Epicuticular wax crystallites were investigated by SEM at different time points after spraying with both surfactants leading to surfactant loads of ca. 1 µg cm^−2^. Directly after surfactant treatment, fine structures of epicuticular wax crystallites appeared blurred and partially disappeared (Fig. 8b, g) compared to the control (Fig. 8a). After 2 to 4 h, epicuticular wax crystallites became more visible and reappeared (Fig. 8c, d, h, i). A complete reappearance (Fig. 8e, j) of the epicuticular waxes was recorded after 6 h and surfaces were no longer different from the control (Fig. 8c, h).

### Time-dependent chemical analysis of C_12_E_4_ residues on leaf surfaces after surfactant treatment

**Fig. 9:**
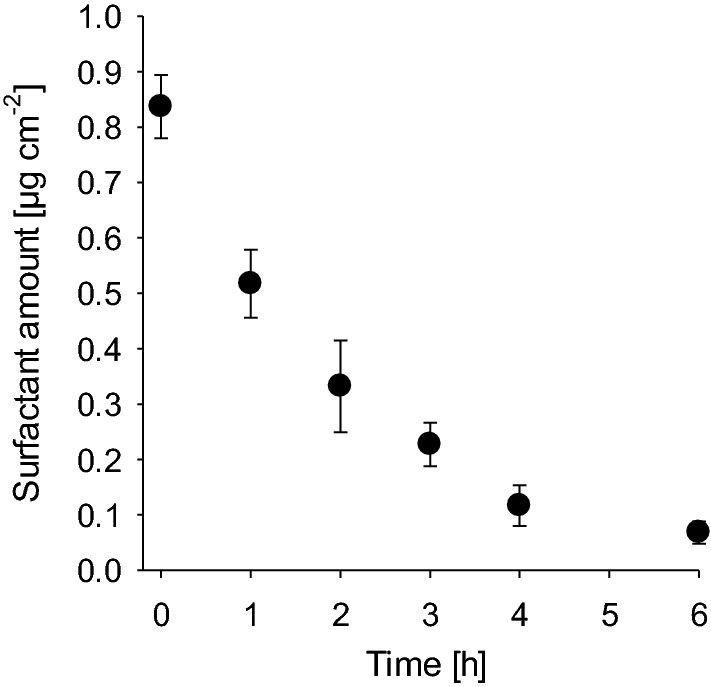
Decreasing amounts (µg cm^−2^) of C_12_E_4_ remaining on the barley leaf surface after spraying with surfactant solution of 0.1% leading to a surface coverage of 1 µg cm^−2^. After 6 h nearly all C_12_E_4_ had disappeared and only 0.07 ± 0.02 µg cm^−2^ of C_12_E_4_ could still be detected on the surface. Data points represent means with standard deviation (*n* ≥ 3)

Chemical analysis by gas chromatography showed that amounts of C_12_E_4_ extracted from leaf surfaces after surfactant spraying with 0.1% solution and drying were continuously decreasing over a time interval of 0 to 6 h (Fig. 9). After 4 h most of the applied C_12_E_4_ (1 µg cm^−2^) had disappeared from the surface. Six hours after application only 0.07 µg cm^−2^ C_12_E_4_ were still detectable on the leaf surfaces.

## Discussion

Spraying leaf surfaces with aqueous 0.1 and 1.0% C_12_E_4_ solutions resulted in reproducible leaf surface coverages very close to 1 and 10 µg cm^−2^ (Fig. 1). With 10% surfactant solution leaf surface coverages were 63 µg cm^−2^. Very different from the hydrophobic barley leaf surface, on the polar glass surfaces the same approach resulted in 1 µg cm^−2^ surfactant coverage only with the 0.1% solution. Surfactant concentrations of 1.0 and 10% resulted in significantly thicker surfactant coverages of 14 and 135 µg cm^−2^ (Fig. 1). Adhesion of the sprayed aqueous surfactant solution on the smooth and polar glass surface is obviously more pronounced compared to the rough and hydrophobic leaf surface. Thus, an optimization of surface coverages of leaf surfaces with surfactants cannot be obtained using a smooth and flat glass surface as an alternative to a rough and hydrophobic leaf surface such as barley.

We investigated whether the surfactants affect basic leaf physiological parameters by measuring photosynthesis and transpiration. Due to their amphiphilic structure, surfactants can disturb membranes by intercalating within phospholipid bilayers, which in turn could lead to changes in membrane fluidity and integrity (Gloxhuber 1974). The electron transport rates in photosynthesis were not affected by both surfactants at a surfactant load of 1 µg cm^−2^ (Fig. 2). At 10 µg cm^−2^, there was a tendency that the yield of photosynthesis was slightly depressed with C_12_E_4_, but not with BrijL4 (Fig. 2). It could be argued that this might be due to the fact that for the monodisperse C_12_E_4_ the total amount inside the leaf exists only of a single molecular species at a fairly high concentration, whereas with the polydisperse BrijL4 the total amount building up in the leaf is given by the sum of the amounts of all the different individual molecular species occurring in BrijL4 at lower concentrations. The highest surfactant coverage fully inhibited photosynthesis within 2 h and killed the leaves (Fig. 2). This can be explained by the stomatal infiltration of the leaves at these extremely high surfactant concentrations, which will directly kill the cells by compromising membrane integrity.

Transpiration was investigated because surfactants have plasticizing effects on the cuticular transport barrier and because it was shown that surfactants, applied at very high surface coverages between 1 and 2 mg cm^−2^, can affect cuticular transpiration (Riederer and Schönherr 1990). Cuticles in this study had an average thickness of 3–5 µm (Schreiber and Schönherr 2009). Assuming a surfactant load of 1 g cm^−3^, a surfactant layer of 1 mg cm^−2^ results in average thickness of 10 µm of surfactants lying on top of the cuticle, which is two- to three-times thicker than the cuticle itself. Cuticles of barley have an average thickness of about 200 nm (Li et al. 2017). Surfactant coverages of 1, 10 and 63 µg cm^−2^, as they were obtained here after drying, lead to a calculated thickness of the corresponding surfactant layers of 10, 100 and 630 nm. At these two lower surfactant coverages, which are on average 2- to 20-times lower than the cuticle thickness, residual cuticular transpiration, measured after stomatal closure, was not significantly affected (Fig. 3). The highest surfactant coverage, which is five-times thicker than the barley cuticle, showed an increase of residual transpiration with both surfactants of about 20% (Fig. 3). Thus, surfactant loads exceeding cuticle thickness may increase cuticular transpiration.

There was also a significant delay in continuous reduction of foliar transpiration compared to control, which was already constant after about 30 min (Fig. 3a, b). With surfactant coverages of 1 and 10 µg cm^−2^ final constant residual cuticular transpiration rates were only obtained between 90 and 150 min, whereas with 63 µg cm^−2^ surfactant coverage residual transpiration did not approach a final constant value (Fig. 3a, b). This delay in reaching the plateau of the residual cuticular transpiration could have been caused by different reasons. It can be explained by the fact that the aqueous surfactants are hygroscopic and thus retain water during dehydration (Asmus et al. 2016). Thus, drying of the applied surfactant solutions takes longer the higher the surfactant coverage is, whereas pure water evaporates within 30 min. The surfactants might also lead to delayed stomatal closure or they might have caused initial increases in cuticular transpiration before they were fully absorbed by the leaves.

Wax amounts and composition were analysed before and after washing off the dried C_12_E_4_ from the leaf surfaces, after leaves had been treated with the three different surfactant amounts (1, 10 and 63 µg cm^−2^). When waxes were extracted directly after surfactants had dried off, amounts were not different from the untreated control with all three coverages (Fig. 4). When wax was extracted from leaves from which the dried C_12_E_4_ was first washed off with water before wax extraction, wax amount and composition was also not different from control at surfactant coverage of 1 µg cm^−2^ (Fig. 4). However, at the two higher coverages of C_12_E_4_ (10 and 63 µg cm^−2^), there was a significant loss of wax (ca. 30%), if surfactants were washed off before wax was extracted. Mainly, amounts of alcohols, alkanes and esters were reduced (Fig. 4). Thus, higher surfactant loads can apparently solubilize wax molecules, which are then removed from the leaf surface when the dried-on surfactants are washed off before wax extraction for chemical analysis. Partial wax removal had previously been reported for artificial wax layers and their interaction with aqueous C_12_E_6_ (Pambou et al. 2018).

Scanning electron microscopic observations give the impression that epicuticular wax structures are damaged and have partially or fully disappeared after treatment with all three surfactant coverages and both surfactants (Fig. 5). This disappearance of wax structures appears to be due to the fact that surfactants are covering the epicuticular wax crystallites. Wax platelets have a height varying between 200 and 1000 nm and the calculated thickness of the surfactant layers at the three different concentrations are between 10, 100 and 630 nm. With increasing thickness of the surfactant layer wax crystallites will continuously disappear within the layer if sprayed with the higher surfactant concentrations. After washing the surfactants off, three-dimensional wax structures fully reappeared at 1 µg cm^−2^ surfactant coverage (Fig. 5b1, b2, e1, e2). Wax crystallites looked completely unaffected and were not different from control surfaces (Fig. 5a). This confirms the analytical data, which showed that wax amounts were not reduced at a surfactant coverage of 1 µg cm^−2^ (Fig. 4). After the application of 10 and 63 µg cm^−2^ with both surfactants, epicuticular wax crystallites were affected (Fig. 5c2, d2, f2, g2). They were reduced in density and their structure had been partially altered. This is in accordance with the analytical data indicating that wax amounts were decreased at these two higher surfactant loads after rinsing (Fig. 4). Contact angle measurements also confirm these observations, because contact angles were again similar to the control after the 1 µg cm^−2^ treatment (Fig. 6), whereas they were significantly lower than the control after the 10 µg cm^−2^ (about 125°) and 63 µg cm^−2^ (about 90°) treatment. This indicates that the three-dimensional structure of the epicuticular wax crystallites was partially disturbed at higher surfactant concentrations when these were rinsed immediately after drying (Fig. 5).

Contact angles of water on leaves, which were fully wettable after treatment with surfactant concentrations of 0.1 and 1%, were increasing with increasing time and reached final values of the controls again (Fig. 7a, c). With a surfactant layer of 1 µg cm^−2^ this rate on increasing contact angles was about 5-times faster (30–60 min) with both surfactants compared to a coverage of 10 µg cm^−2^ (300 min). Obviously, surfactants deposited on the leaf surface are diffusing with time across the cuticle into the leaf tissue. Thus, the thickness of the surfactant layer on the outer surface of the cuticle must decrease with time and consequently contact angles must increase again (Fig. 7a, c). Hence, by only measuring the increase of the contact angles over time, cuticular uptake of foliar applied surfactants can indirectly be shown. Initial recovery rates (angle in ° per time) can be calculated from the slopes and recovery was ca. three times slower at a ten times higher surfactant load (Fig. 7b, d). It can also be discussed that the full reappearance of the high contact angles of about 140° could potentially also occur with residual amounts of surfactants still remaining on the leaf surface. In case of Cassie–Baxter wetting, the droplets of pure water would be sitting on the tips of the reappearing hydrophobic wax crystallites, whereas potentially remaining surfactant molecules would be located at the base between the wax crystallites and thus surfactants and pure water would not be in direct contact but spatially separated from each other by an air pocket (Schulte et al. 2011).

It is evident, that this process of contact angle recovery is significantly delayed with the ten times thicker surfactant layer (10 µg cm^−2^ corresponding to 100 nm; Fig. 7a, c). Leaves stay fully wettable for about 3 h, before contact angles recover, whereas recovery started within a few minutes with the low surfactant load (1 µg cm^−2^ corresponding to 10 nm). As long as water droplets are sitting on the outermost layer of the deposited surfactant layers, leaves stay fully wettable. With the thicker surfactant coverage, it just takes much longer until the hydrophobic, non-wettable waxy leaf surface is reappearing and gets again in direct contact with the water droplet. Only then original final contact angles are reappearing. Thus, different from washing off the surfactants directly after drying, which affects leaf surface structure and wetting at 10 µg cm^−2^ surfactant coverage (Figs. 5, 6), this is not the case when the surfactants are staying on the leaf surface and disappearing over time (Fig. 7). Then leaf surface wetting is not affected and can fully recover (Fig. 7).

**Fig.10:**
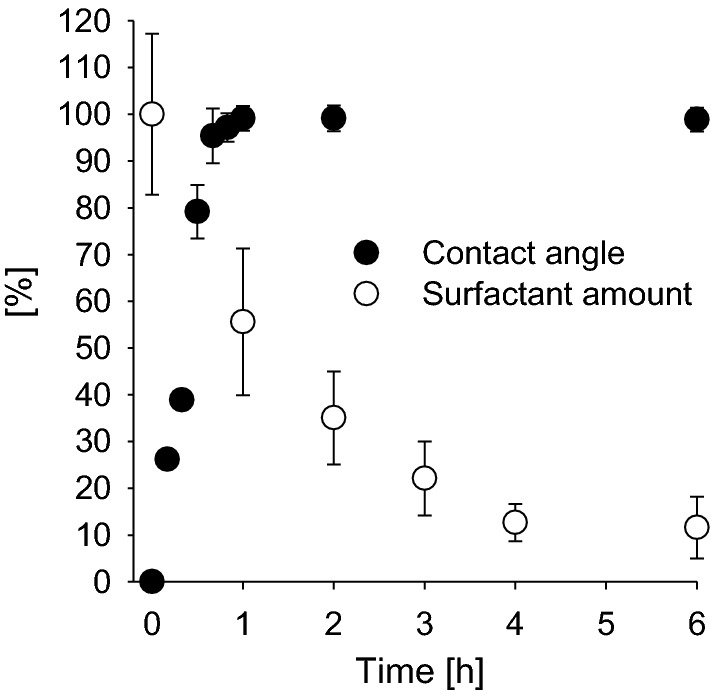
Time-dependent recovery of the relative contact angle (%) on the barley leaf surface (black circles) in comparison to the disappearance (%) of C_12_E_4_ (white circles) form barley leaf surface. Data points represent means with standard deviation (*n* ≥ 3)

The time-dependent investigation of the surfactant-treated leaf surfaces using scanning electron microscopy confirms this. Micrographs show that in parallel to the contact angle recovery, epicuticular wax crystallites were fully reappearing with both surfactants at 0.1% solutions (Fig. 8). When plotting the contact angle recovery and the disappearance of C_12_E_4_ from the leaf surface is plotted as percentage values, it become obvious that only about 50% of the initial surfactant amount (1 µg cm^−2^) have disappeared after 60 min. However, at this time contact angles were already fully recovered (Fig. 10). This might be due to residual amounts of surfactants still located at the very bottom on the leaf surface between the single wax platelets, whereas the water droplet for contact angle measurements will be sitting on the tips of the hydrophobic and rough epicuticular wax crystallites. Alternatively, this discrepancy could also be due the fact, that the analytical quantification overestimates the amounts of surfactant. It cannot be excluded that surfactants are not only extracted from the leaf surface, but to some extent also from the cuticle interior or the outer epidermal cell wall, whereas contact angles are affected only by the outermost atomic layer of a surface (Holmes-Farley et al. 1988). This fraction of surfactants lying deeper in the cuticle will not contribute anymore to a decrease of the contact angle on the leaf surface, but it would contribute to the analytical quantification of the surfactants.

## Conclusion

Our study shows that non-ionic surfactants sprayed at surface loads of 1 and 10 µg cm^−2^ to leaf surfaces of barley leading to 100% wetting rapidly diffuse through the cuticle and are eliminated from the leaf surface. We found no significant differences between the effects of the monodisperse (tetraethylene glycol monododecyl ether) and the polydisperse alcohol ethoxylate (BrijL4). Non-wettability of leaves recovered fully and three-dimensional structure of epicuticular wax crystallites were reconstituted. We conclude that leaf surface wetting properties (Fig. 7) and microstructures (Fig. 8) are not significantly or irreversibly altered at 1 and 10 µg cm^−2^ surfactant loads, representing realistic coverages as they are found during spray application in the field. However, at significantly higher surfactant loads (63 µg cm^−2^) leaves were rapidly killed within a couple of hours and wax structure was clearly altered. It can also be postulated that subsequent surfactant applications will not necessarily change leaf surface properties as long as there is enough time for surfactant diffusion into the leaf between subsequent applications. However, the situation might be very different with surfactants staying on the leaf and not diffusing into the leaf, e.g. due to their size and/or polarity. This is currently under investigation, applying this method of measuring time-dependent changes of contact angles after application of further surfactants varying in size and polarity.

### *Author contribution statement*

LS obtained the grants to support this study. LS, JB and VZ designed the study and planned the experiments. JB, VZ and YM conducted the experiments. All authors analysed the data. JB and LS wrote the manuscript. All authors read and approved the manuscript.

## Data Availability

All data generated or analysed during this study are included in this published article.
